# Supplementary material for: Pharmacogenetics of Donepezil and Memantine in Healthy Subjects
Source: J Pers Med. 2022 May 13;12(5):788. doi: 10.3390/jpm12050788 (PMC9145014; doi:10.3390/jpm12050788)
Supplement: Supplementary file 1 [file jpm-12-00788-s001.zip › jpm-1702195-supplementaryS1.pdf]

**Table S1.** Association between the polymorphism and Alzheimer's disease or drugs used for their treatment included in the present study.

| Gene | Variant | Name | Association with Alzheimer/Donepezil–Memantine | References |
|---|---|---|---|---|
| *ABCB1* | rs1128503 (*C1236T*) | ATP Binding Cassette subfamily B member 1 | The ABCB1 C1236T, G2677T/A, and C3435T SNPs influence BBB P-glycoprotein function. ABCB1 is a transporter of donepezil | [8] |
| | rs1045642 (*C3435T*) | | | |
| | rs2032582 (*G2677A*) | | | |
| | rs2032582 (*G2677T*) | | | |
| | rs10248420 | | | |
| | rs10276036 | | | |
| | rs10280101 | | | |
| | rs11983225 | | | |
| | rs12720067 | | | |
| | rs3842 | | | |
| | rs4148737 | | | |
| | rs4728709 | | | |
| | rs7787082 | | | |
| *ADRA2A* | rs1800544 | Adrenoceptor alpha 2A | The mechanistic gene of anti-Parkinsonian drugs such as apomorphine. It is also involved in AD | [56] |
| *APOC3* | rs4520 | Apolipoprotein C-III | | [57] |
| | rs5128 | | | |
| *BDNF* | *rs6265* | Brain-derived neurotrophic factor | Memantine and donepezil induce BDNF synthesis. BDNF is involved in AD | [48,53,54] |
| *COMT* | rs13306278 | Catechol O-methyltransferase | It is involved in AD pathology | [44] |
| | rs4680 | | | |
| *CYP1A2* | rs2470890 (*1B) | Cytochrome P450 family 1 subfamily A member 2 | Memantine is a weak inhibitor of CYP1A2. In human liver microsomes, memantine does not affect CYP1A2 activity | [58] |
| | rs2069514 (*1C) | | | |
| | rs762551 (*1F) | | | |
| *CYP2A6* | rs28399433 | Cytochrome P450 family 2 subfamily A member 6 | Memantine is a weak inhibitor of CYP2A6. In human liver microsomes, memantine decreases CYP2A6 activity | [55] |
| *CYP2B6* | rs3211371 (*5) | Cytochrome P450 family 2 subfamily B member 6 | Memantine is a strong inhibitor of CYP2B6. In human liver microsomes, memantine inhibits CYP2B6 activity | [55] |
| | rs3745274 (*9) | | | |
| | rs2279343 | | | |
| | rs2279345 | | | |
| | rs4803419 | | | |
| *CYP2C19* | rs12248560 (*17) | Cytochrome P450 family 2 subfamily C member 19 | Memantine is a weak inhibitor of CYP2C19. In human liver microsomes, memantine decreases CYP2C19 activity | [55] |
| | rs4244285 (*2) | | | |
| | rs4986893 (*3) | | | |
| | rs28399504 (*4) | | | |
| *CYP2C8* | rs11572103 (*2) | Cytochrome P450 family 2 subfamily C member 8 | The substrate of selegiline, an anti-Parkinsonian drug | |
| | rs10509681 (*3) | | | |
| | rs1058930 (*4) | | | |
| *CYP2C9* | rs1799853 (*2) | Cytochrome P450 family 2 subfamily C member 9 | Donepezil is a substrate of CYP2C9 | [59] |
| | rs1057910 (*3) | | | |
| **Gene** | **Variant** | **Name** | **Association with Alzheimer/Donepezil–Memantine** | **References** |
| *CYP2D6* | rs1065852 (*10) | Cytochrome P450 family 2 subfamily D member 6 | Donepezil is a substrate of CYP2D6. There is a drug label for donepezil dose adjustment based on CYP2D6 genotype | [8] |
| | rs5030865 (*14) | | | |
| | rs28371706 (*17) | | | |
| | rs35742686 (*3) | | | |
| | rs3892097 (*4) | | | |

| | | | | |
|---|---|---|---|---|
| | rs28371725 (**41*) | | | |
| | rs5030655 (**6*) | | | |
| | rs5030867 (**7*) | | | |
| | rs5030865 (**8*) | | | |
| | rs5030656 (**9*) | | | |
| *CYP3A4* | rs55785340 (**2*)<br>rs35599367 (**22*)<br>rs4646438 (**6*) | Cytochrome P450 family 3 subfamily A member 4 | Memantine is a weak inhibitor of CYP3A4. In human liver microsomes, memantine does not affect CYP3A4 activity. In addition, CYP3A4 is a major substrate of donepezil | [8] |
| *CYP3A5* | rs776746 (**3*)<br>rs10264272 (**6*) | Cytochrome P450 family 3 subfamily A member 5 | The substrate of anti-Parkinsonian drugs such as levodopa | [8] |
| *CYP4F2* | rs2108622 | Cytochrome P450. family 4. subfamily F. polypeptide 2 | The hepatic CYP450 enzyme system does not majorly contribute to the metabolism of this drug | |
| *DRD2* | rs1799732<br>rs1800497<br>rs6277 | dopamine receptor D2 | Target of memantine | [52] |
| *DRD3* | rs6280 | dopamine receptor D3 | Target of memantine | [60] |
| *EPHX1* | rs1051740<br>rs2234922 | Epoxide hydrolase 1. microsomal (xenobiotic) | Show differential expression in AD | [61] |
| *HTR2A* | rs6313<br>rs6314<br>rs7997012 | serotonin receptor | HTR2A is a target for donepezil | [62] |
| *HTR2C* | rs1414334<br>rs3813929<br>rs518147 | | HTR2C is mildly involved in AD | |
| *PTGS2* | rs20417 | Prostaglandin-endoperoxide synthase 2 (prostaglandin G/H synthase and cyclooxygenase) | Associated with AD risk | [50] |
